# Viromes and surveys of RNA viruses in camel-derived ticks revealing transmission patterns of novel tick-borne viral pathogens in Kenya

**DOI:** 10.1080/22221751.2021.1986428

**Published:** 2021-10-17

**Authors:** You Zhang, Ben Hu, Bernard Agwanda, Yaohui Fang, Jun Wang, Stephen Kuria, Juan Yang, Moses Masika, Shuang Tang, Jacqueline Lichoti, Zhaojun Fan, Zhengli Shi, Sheila Ommeh, Hualin Wang, Fei Deng, Shu Shen

**Affiliations:** a State Key Laboratory of Virology and National Virus Resource Centre, Wuhan Institute of Virology, Chinese Academy of Sciences, Wuhan, People’s Republic of China; b CAS Key Laboratory of Special Pathogens and Biosafety, Wuhan Institute of Virology, Chinese Academy of Sciences, Wuhan, People’s Republic of China; c University of Chinese Academy of Sciences, Beijing, People’s Republic of China; d Department of Zoology, National Museums of Kenya, Nairobi, Kenya; e Institute For Biotechnology Research (IBR), Jomo Kenyatta University of Agriculture and Technology (JKUAT), Nairobi, Kenya; f Department of Medical Microbiology, University of Nairobi Nairobi, Kenya; g Directorate of Veterinary Services, State Department of Livestock, Ministry of Agriculture, Livestock, Fisheries and Irrigation, Nairobi, Kenya

**Keywords:** Ticks, virome, tick-borne viruses, camels, viral transmission correlation

## Abstract

Tick-borne viruses (TBVs) capable of transmitting between ticks and hosts have been increasingly recognized as a global public health concern. In this study, *Hyalomma* ticks and serum samples from camels were collected using recorded sampling correlations in eastern Kenya. Viromes of pooled ticks were profiled by metagenomic sequencing, revealing a diverse community of viruses related to at least 11 families. Five highly abundant viruses, including three novel viruses (Iftin tick virus, Mbalambala tick virus [MATV], and Bangali torovirus [BanToV]) and new strains of previously identified viruses (Bole tick virus 4 [BLTV4] and Liman tick virus [LMTV]), were characterized in terms of genome sequences, organizations, and phylogeny, and their molecular prevalence was investigated in individual ticks. Moreover, viremia and antibody responses to these viruses have been investigated in camels. MATV, BLTV4, LMTV, and BanToV were identified as viral pathogens that can potentially cause zoonotic diseases. The transmission patterns of these viruses were summarized, suggesting three different types according to the sampling relationships between viral RNA-positive ticks and camels positive for viral RNA and/or antibodies. They also revealed the frequent transmission of BanToV and limited but effective transmission of other viruses between ticks and camels. Furthermore, follow-up surveys on TBVs from tick, animal, and human samples with definite sampling relationships are suggested. The findings revealed substantial threats from the emerging TBVs and may guide the prevention and control of TBV-related zoonotic diseases in Kenya and in other African countries.

## Introduction

Ticks are common hematophagous arthropods that can infest and spread pathogenic bacteria, protozoans, rickettsia, and viruses to human and animal hosts by sucking their blood [1–3]. The significance of tick-borne viral diseases (TBVDs) in human health has been increasingly recognized in recent decades. Newly emerging tick-borne viral pathogens causing febrile human diseases, such as severe fever with thrombocytopenia syndrome virus [4], Heartland virus [5], Alongshan virus [6], Jingmen tick virus [7], and Songlin virus [8], have been identified in North American and East Asian countries. Furthermore, the re-emergence and continuous spread of known tick-borne viruses (TBVs), such as Crimean – Congo hemorrhagic fever virus (CCHFV), Kyasanur Forest virus, Alkhurma hemorrhagic fever virus, Powassan virus, Deer tick virus, and African swine fever virus, positively correlate with the increasing incidence of TBVDs in humans and animals [9].

Recently, many undefined viruses were discovered in ticks, promote our knowledge of the biodiversity and evolution of viruses vectored by ticks. Viromes of different tick species in various hosts collected in China [10], Australia [11], Brazil [12], Turkey [13], and the United States [14,15] have suggested a worldwide distribution and high prevalence of zoonotic TBVs. However, studies on TBVDs in African countries are limited. Epidemiological studies have identified viral pathogens related to severe human diseases in Africa, such as CCHFV [16] and tick-borne encephalitis virus (TBEV) [17]. These human disease-causing viruses have high seroprevalence in domestic animals and livestock farmers in Uganda [18], Tunisia [19], Senegal [20], Sudan [21], and Kenya [22]. Kenya is a sub-Saharan country predicted to have a higher risk of human CCHF infection [23,24]. Although a few human cases of CCHFV infection have been reported, with the first case recorded in 2002 in Kenya [25], the potential risks of CCHF outbreaks and epidemics in Kenya should not be underestimated as a high seroprevalence in humans and high infection rates of CCHFV in ticks have been reported in recent decades [16,17,22,26,27]. Although no cases of infection have been reported, TBVs such as Dhori virus and Dugbe virus (DUGV), associated with human and livestock diseases, have been isolated from ticks in Kenya [28]. This finding suggests that the Kenyan population has a significant risk of developing TBVDs. In addition, other novel viral pathogens might not have been recognized or fully characterized due to technological limitations. For instance, the Kupe virus, a novel virus closely related to DUGV isolated from ticks in Kenya in 1999, was only fully sequenced and genetically characterized in 2009 when the appropriate technology became available [29–31]. To date, the potential health threats from transmission and infection of TBVs among humans and domestic animals in Kenya are poorly understood. Therefore, profiling viruses in ticks and investigating the substantial contacts between ticks and human or animal hosts is urgently needed to better understand the viral sphere vectored by ticks and identify the potential viral pathogens in Kenya.

In this study, ticks of three *Hyalomma* species were collected from camels in eastern Kenya in 2018. Viromes of tick pools were profiled via metagenomic sequencing aimed at identifying the baseline viruses vectored by ticks and characterizing their biodiversity and evolution. Furthermore, the potential virus transmission between ticks and camels was investigated by surveying the virus prevalence among individual ticks and related camels from whom ticks were collected and serological exposure to these viruses among the camels. Finally, the viral transmission patterns between ticks and camels were investigated and discussed based on the results of molecular prevalence and serological exposure to viruses and correlations between viral RNA-positive ticks and camels. These findings may shed light on the diversity of TBVs in Kenya and reveal the potential risks of TBV transmission between ticks and animal hosts, which could promote a better understanding of TBVs and guide disease prevention and control in African countries such as Kenya.

## Materials and methods

### Collection of ticks and serum samples from camels

**Figure 1.**
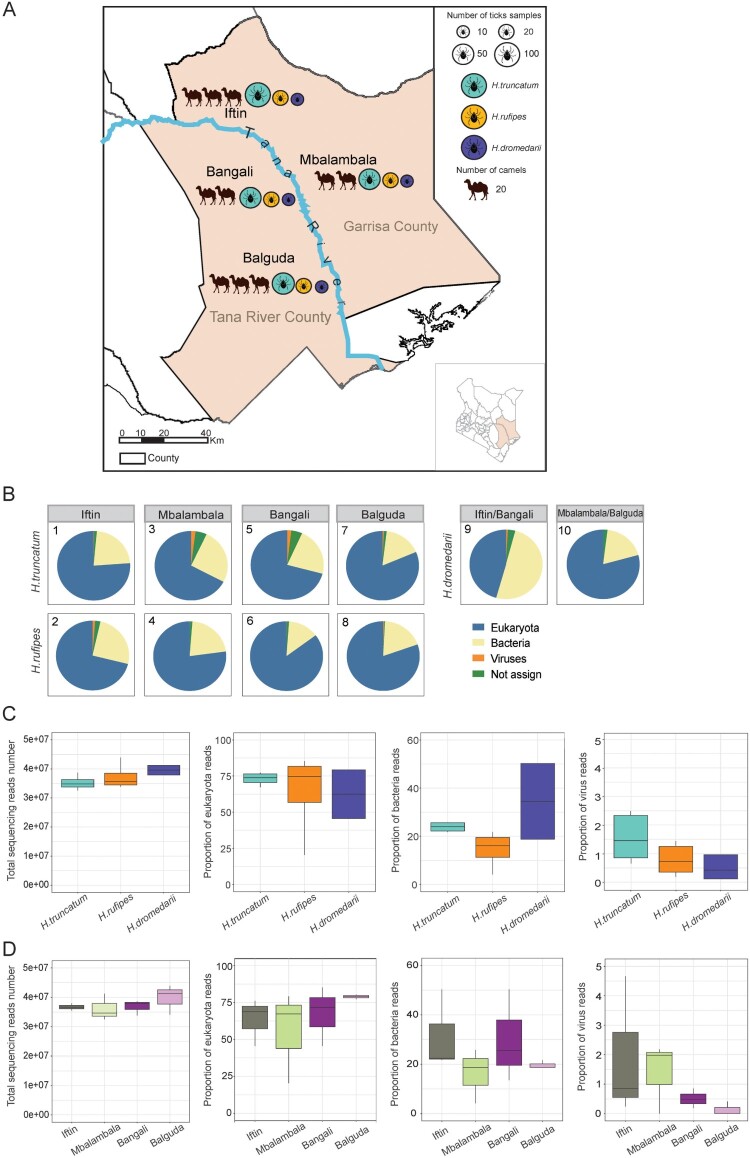
Tick collection in eastern Kenya and metagenomics of ticks according to species and locations. (A) The map of Garrisa and Tana River County, Kenya, shows where tick and camel serum samples were collected from. Tick species and the number of ticks and camels are also shown on the map with different colours and sizes. (B) The proportions of the numbers of reads assigned to eukaryotes, bacteria, and virus and number of unassigned reads in each of the 10 pools are shown in pies. (C) Comparison of the number of total reads and proportions of eukaryotic, bacterial, and viral reads from the three tick species. (D) Comparison of the numbers of total reads and proportions of eukaryotic, bacterial, and viral reads from the four sampling locations.

A total of 396 ticks were collected from 200 camels from four locations (Iftin, Mbalambala, Balguda, and Bangali) in Kenya in 2018 (Figure 1). Each tick was numbered according to their sampling relationships with the camels from whom the serum samples were collected. Tick species were first classified using morphological methods by a professional technician and were further confirmed based on the mitochondrial cytochrome c oxidase subunit I gene sequence (Supplementary Methods). Data on the locations, species, and numbers of tick samples were recorded (Table S1).

### Metagenomic sequencing

To investigate the diversity and richness of viromes harboured in ticks, we established 10 tick sequencing pools according to the different locations and tick species (Table S2). Total RNA was extracted from the tick pools, as previously described [32]. RNA from each pool (3 μg) was subjected to RNA sequencing (RNA-seq) using a HiSeq 3000 sequencer according to the manufacturer’s instructions (Illumina, San Diego, CA, USA). The reads generated from each pool were evaluated, filtered, and assembled to obtain virus-related transcripts (Supplementary Methods). Transcripts per million (TPM) was used as a measure to estimate and characterize the abundance of mapped viral sequences to avoid any bias caused by unequal gene length or sequencing depth between different libraries [33,34]. TPM values on the log10 scale were used to show the normalized abundance of each viral contig in the heatmap. The coverage rates and sequence identities of the viral-related sequences compared to those of reference viruses were also calculated using a heatmap. The assigned viral species names were arranged by viral family annotation via DIAMOND and BLASTx comparisons. In addition, a second round of sequencing was performed using the remaining RNA from pool 6. The Bangali torovirus (BanToV) genome was obtained by combining the data from two rounds of sequencing.

All RNA-Seq data in this study were deposited in the NCBI Sequence Read Archive database (BioProject accession number: PRJNA732268). All virus genome sequences identified in this study were deposited in GenBank (accession numbers: MW561965 to MW561977).

### Phylogeny of viruses and data analysis

The putative open reading frame (ORF) sequences of viral proteins were used to predict their function using PSI-BLAST. Phylogenetic trees were constructed using full-length amino acid (aa) sequences of putative proteins or ORFs, including Mbalambala tick virus (MATV) and Iftin tick virus (IFTV) (RNA-dependent RNA polymerase [RdRp] and nucleoprotein [NP]), Liman tick virus (LMTV) (RdRp, glycoprotein [GP], and NP), Bole tick virus 4 (BLTV4) (polyprotein), and BanToV (1a, S1, S2, M, HE, and NP); the full-length genome sequence of BanToV; and the protein sequences or genome sequences of related viruses of the families *Phenuivirudae*, *Flaviviridae*, *Chuviridae*, and *Tobaniviridae*. Sequence alignments were performed using MEGA 7.0.26 (https://www.megasoftware.net). The best-fit model selection for aa substitution was processed prior to the construction of the maximum likelihood phylogenetic tree with 1,000 bootstrap replications.

Heatmaps presenting virus abundance in each pool and the sequence identities and percentages of coverage to corresponding reference viruses were generated using TBtools (https://github.com/CJ-Chen/TBtools/releases). Quantification of selected viruses from individual ticks and camels was performed using the ggplot2 package in R studio [35]. The landscape of viral transmission correlations between ticks and camels was plotted and adjusted using d3.js (https://d3js.org).

### Molecular detection of viral RNA in tick individuals and serum samples from camels

After performing sequencing analysis, the remaining tick individuals (241 *H. truncatum* ticks, 78 *H. rufipes* ticks, and 17 *H. dromedarii* ticks) were used to investigate the prevalence of IFTV or MATV, BLTV4, LMTV, and BanToV using the PCR primers and probes designed according to viral RdRp or polyprotein sequences (Table S3). A bead-based assay was performed to allow multiple detection of viruses in one reaction according to a previous study [36] and detect viral RNA in each tick (Supplementary Methods). qRT-PCR was performed to determine the viral RNA copies in individual ticks and camel serum samples using the same primers and probes as those utilized in bead-based multiplex assays (Supplementary Methods).

### Detection of antibody levels against viruses

Luciferase immunoprecipitation system (LIPS) assays were performed to detect antibody responses to viruses in camel serum samples, as previously described [37]. The NP of MATV (1098 bp), LMTV (1374 bp), BanToV (636 bp), and GP of BLTV4 (477 bp) were cloned into the plasmid pREN2 (provided by Linfa Wang [Duke University, Singapore] and Peng Zhou [Wuhan Institute of Virology, Chinese Academy of Sciences, China]) in fusion with the Renilla luciferase (Ruc) and a Flag tag. All recombinant plasmids were verified by Sanger sequencing and then transfected into HEK293 T cells using Lipofectamine 3000 (Thermo Fisher Scientific, USA). The protein expression in transfected cells was validated by western blot using anti-Flag antibody (AB18230, Abcam, Cambridge, UK) (data not shown). The light unit (LU) of each Ruc-viral antigen was measured using the Renilla-Lumi^™^ Luciferase Assay Kit (Beyotine, China) on a GloMax 20/20 Luminometer (Promega, USA). LIPS assays were performed to screen for multiplex viral antibodies in camel serum samples (Supplementary Methods).

### Ethics statement

This study was performed in accordance with the institutional and national guidelines for the care and handling of the animals. This study received clearance from the director of veterinary services and the county governments of Garrisa and Tana River.

## Results

### Collection of ticks from camels in eastern Kenya

A total of 396 tick samples were collected from 200 camels in Garrisa County (Iftin and Mbalambala) and Tana River County (Bangali and Balguda), Kenya, in 2018 (Figure 1A and Table S1). The ticks were identified as *H. truncatum* (65.90%), *H. rufipes* (24.74%), and *H. dromedarii* (9.36%) according to their morphology and further confirmed by phylogenetic analysis (Figure S1). Ten pools of ticks were grouped according to their sampling locations and species, with each group consisting of 5–10 ticks (Table S2). RNA was purified from each pool and subjected to RNA-seq, generating comparable amounts of total reads (33,808,271–43,927,631; Table S2). The non-tick reads were assigned to eukaryotes (81.71% – 41.14% of the total reads), bacteria (17.62%–55.34%), and viruses (0.1%–0.34%) by BLAST comparison, presenting proportions similar to that of the taxonomy in terms of the read numbers, except the pool containing *H. dromedarii* from Iftin and Mbalambala, which had more bacterial reads (55.34%) (Figure 1B). When combining the reads together, the three tick species had comparable numbers of total reads (3.22–4.39 × 10^7^). The proportions of eukaryote, bacterial, and viral reads were more variable in pools containing *H. dromedarii*. Although the proportions of reads assigned to eukaryotes, bacteria, and viruses from pools varied, and significant differences were not observed among the three tick species (Figure 1C). Similarly, the total number of reads was comparable among ticks from the four sampling locations. Although the proportions of reads associated with eukaryotes, bacteria, and viruses varied according to their sampling locations, differences among Iftin, Mbalambala, Bangali, and Balguda were not significant (Figure 1D).

### Profiling viral diversity among ticks from camels in Kenya

**Figure 2.**
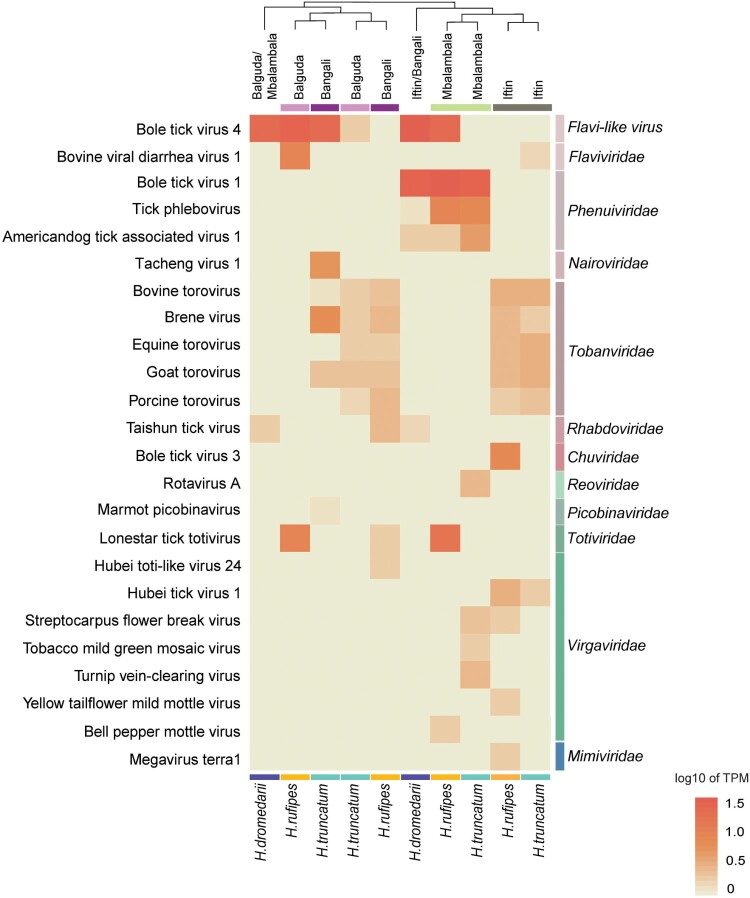
Normalized abundance of viral species in tick pools. Comparative abundances of different viral species reads are normalized by TPM counts, and the log10 scale of TPM is calculated for hierarchical clustering using the Euclidean distance matrix in the heatmap. The viral species names identified by taxonomic annotation using BLASTx are sorted by viral family in the heatmap. The sampling locations and species of ticks are marked above and below the heat map.

Data from the ten tick pools were subsequently used to profile viromes in terms of tick species and sampling locations. Virus-related sequences from the tick pools (Table S4) were annotated and assigned to viruses belonging to at least 11 families, including *Flaviviridae*, *Tobamviridae*, *Virgaviridae*, *Phenuiviridae*, *Nairoviridae*, *Rhabdoviridae*, *Chuviridae*, *Reoviridae*, *Totiviridae*, *Picobinaviridae*, and *Mimiviridae*. The abundance of viral species, as shown in a heatmap (Figure 2), indicated that viromes hosted by camel-derived ticks might be correlated with their geographic distribution (Figure 2). Iftin and Bangali had richer and more diverse toroviruses (family *Tobanviridae*) than Mbalambala and Balguda. Viral sequences belonging to the family *Phenuiviridae* were identified mainly from tick pools in Mbalambala. A high abundance of flavivirus (bovine viral diarrhea virus 1) was found in the *H. rufipes* tick pool, while flavi-like virus (BLTV4) sequences were widely distributed in the tick pools from Balguda, Bangali, and Mbalambala. Viral sequences related to the family *Virgaviridae* were found in tick pools from Iftin and Mbalambala. Moreover, flavi-like virus and torovirus sequences shared high sequence identity with their reference viral sequences, while flavi-like virus, phenuivirus, and chuvirus sequences showed high levels of coverage compared to full-length genomic sequences of their respective reference viruses (Figure S2). The presence of these viruses in the corresponding pool was confirmed by nested RT–PCR and Sanger sequencing (data not shown).

### Viral genome organization and phylogeny characterization

The complete genome sequences or ORFs of five viruses were obtained, including three novel viruses belonging to the families *Phenuiviridae* and *Tobanviridae*, and new strains of two viruses belonging to the family *Chuviridae* and flavi-like virus (Table 1). The novel viruses and new strains were named according to their location, tick species, and year in which the virus-positive tick samples were collected.

**Table 1.** Detailed information of novel viruses and new strains of complete genome sequences identified by RNAseq in this study

Event	Family	Virus	Novel virus	Strains	Genome length (nt)	Closest virus	% ORF identity (aa)	Abundance (TPM)
1	*Phenuiviridae*	Iftin tick virus	Y	*H. dromedarii/*2018/01	6474 (L segment)	Bole tick virus1 (strain 17-L2)	88 (RdRp)	13.14
	* *				1627 (S segment)		83 (NP)	
2	*Phenuiviridae*	Mbalambala tick virus	Y	*H. truncatum*/2018/01	6477 (L segment)	Xinjiang tick phlebovirus (strain YDL)	80 (RdRp)	8.98
					1697 (S segment)		65 (NP)	
			* *	*H. rufipes*/2018/02	6481 (L segment)	Xinjiang tick phlebovirus (strain YDL)	80 (RdRp)	32.54
					1725 (S segment)		65 (NP)	
			* *	*H. dromedarii*/2018/03	6475 (L segment)	Xinjiang tick phlebovirus (strain YDL)	80 (RdRp)	14.62
					1699 (S segment)		73 (NP)	
3	Flavi-like virus	Bole tick virus 4	N	Bangali/*H. truncatum*/2018	16223	Bole tick virus 4 (strain BLP-1)		
	92 (Polyprotein)	2.89						
				Mbalambala /*H. rufipes*/2018	16257		92 (Polyprotein)	16.85
				Iftin/*H. dromedarii*/2018	16225		94 (Polyprotein)	32.88
4	*Chuviridae*	Liman tick virus	N	Iftin/*H. rufipes*/2018	11186	Liman tick virus (Rus/*H. anatolicum*/Astrakhan/mon1/2019)	90 (RdRP)	7.25
	* *						90 (GP)	
	* *						92 (NP)	
5	*Tobaniviridae*	Bangali Torovirus	Y	*H. rufipes*/2018	28847	Brene virus (strain P138/72)	75 (1a)	3.25
							87 (S)	1.34
							90 (M)	2.35
							88 (HE)	4.68
							71 (NP)	1.46

#### Phenuiviridae: IFTV and MATV

**Figure 3.**
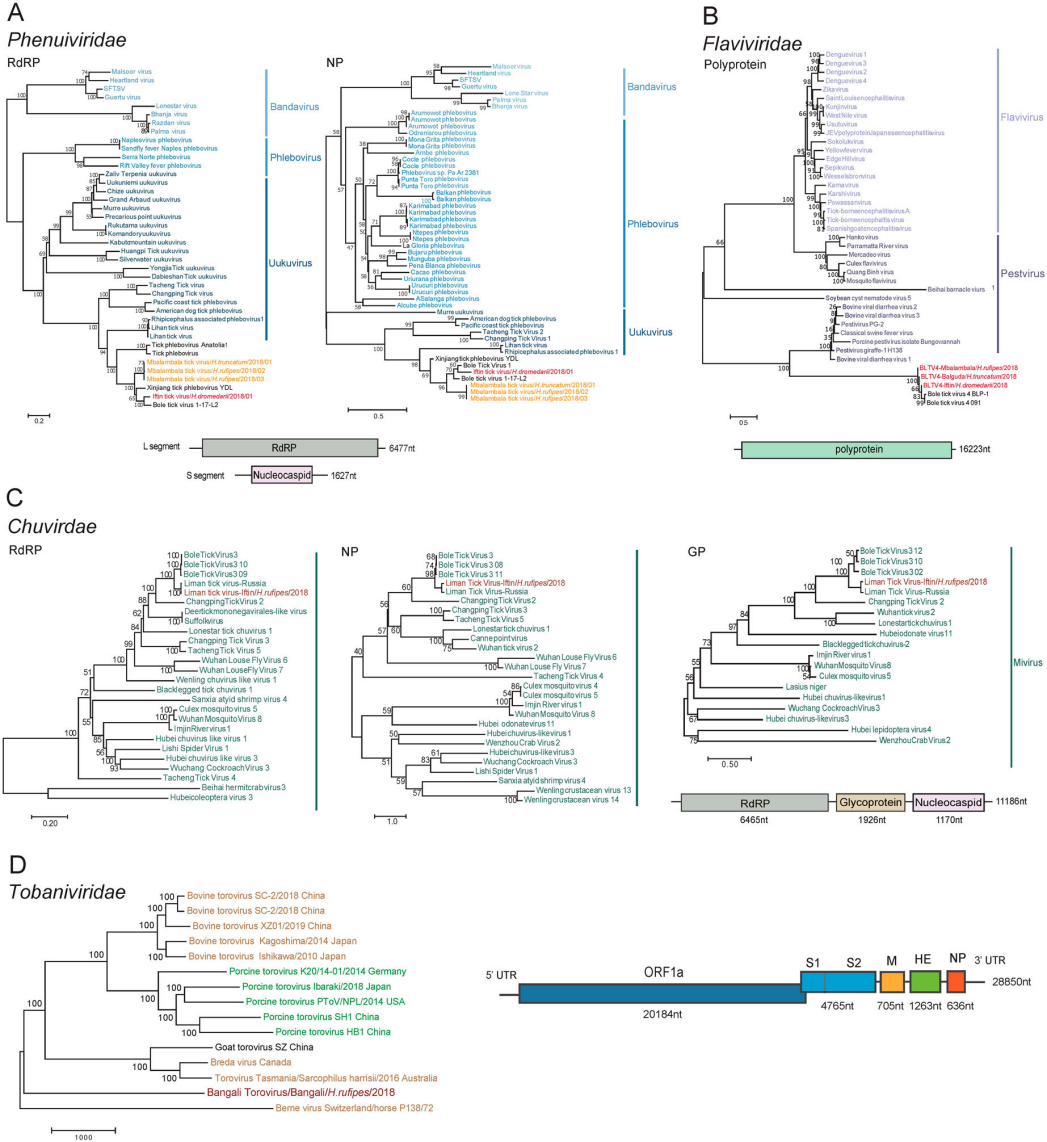
Phylogenetic relationships and genome organizations of the viruses identified from ticks. Phylogenetic trees of the *Phenuiviridae* (A), *Flaviviridae* (B), and *Chuviridae* (C) families including MATV, IFTV, BLTV4, and LMTV are constructed based on the full length of the aa sequence of corresponding viral proteins, respectively, and the tree of *Tobaniviridae* family including BanToV (D) is constructed using the full length of the viral genome. The viruses identified in this study are shown in red font. Viruses belonging to different groups are labelled with different colours. The genome organization of each virus is presented in a schematic diagram below or outside the trees.

IFTV and MATV have been identified as novel viruses. The L and S segments of IFTV were identified in the *H. dromedarii* pools from Iftin and Bangali, whereas the L and S segments of MATV were identified in the pools of *H. truncatum* and *H. rufipes* from Mbalambala and in the pool of *H. dromedarii* from Iftin and Bangali (Table 1). IFTV is most closely related to Bole tick virus 1 (BLTV1) strain 17-L2, while MATV is most closely related to the Xinjiang tick phlebovirus (XJTPV) strain YDL. Similar to the genome organization of the reference viruses, the IFTV and MATV S segments encoded the ORFs of NP (316 and 362 aa, respectively), while the L segments encoded RdRp (2145 and 2149 aa, respectively). IFTV had 88% and 83% aa identity of RdRp and NP of BLTV1, respectively. The three MATV strains shared 80% and 73% identities with the RdRp and NP of XJTPV, respectively. Moreover, IFTV and MATV shared 89% and 80% aa identities with the S and L segments, respectively. Phylogenetic analyses showed that IFTV and MATV were two novel viruses belonging to the family *Phenuiviridae*. As expected, IFTV clustered with BLTV1, whereas the three MATV strains clustered with XJTPV. They formed a clade that was very close to the genus *Uukuvirus* and was not assigned to any genus (Figure 3A).

#### Flavi-like virus: BLTV4

BLTV4 is a flavi-like virus whose genome organization is similar to that of flaviviruses, but cannot be assigned to any genus of the family *Flaviviridae*. BLTV4-related viral sequences were identified in six tick pools, including *H. truncatum* from Bangali and Balguda, *H. rufipes* from Mbalambala and Balguda, and *H. dromedarii* from Iftin and Bangali. Three new strains of BLTV4 were identified and designated as strain Bangali/*H. truncatum*/2018, strain Mbalambala/*H. rufipes*/2018, and strain Iftin/*H. dromedarii*/2018 according to the locations of the tick pools from which they were obtained. The BLTV4 genome sequence was 16,200 nucleotides (nt) in length and contained one ORF that encoded a polyprotein. The three BLTV4 strains shared 88%–91% nt similarity and 92%–94% aa similarity with the reference strain BLP-1 (Table 1). Phylogenetic analysis showed that all BLTV4 strains formed a clade that was close to the genus *Pestivirus* of the family *Flaviviridae* (Figure 3B).

#### Chuviridae: LMTV

A new LMTV strain (Iftin/*H.rufipes*/2018) was identified in the pool of *H. rufipes* in Iftin. This new strain has a circular ssRNA virus of 11,100 nt in length and three ORFs that encode RdRp, (6465 nt, 2155 aa), GP (1926 nt, 651 aa), and NP (1170 nt, 458 aa). The new strain shared high sequence identity (90%, 90%, and 92% aa identities with RdRp, GP, and NP, respectively) with the reference strain Rus/*H. anatolicum*/Astrakhan/mon1/2019. Phylogenetic analysis using the RdRp, GP, and NP aa sequences demonstrated that the LMTV belongs to the genus *Mivirus* of the family *Chuviridae* and is most closely related to Bole tick virus 3 and Changping tick virus 2 (Figure 3C).

#### Tobaniviridae: bantov

Torovirus-related reads were identified with varied identities to the reference toroviruses in six tick pools of *H. trumcatum* and *H. rufipes* ticks from Iftin, Bangali, and Balguda (Table S4, Figure S2), suggesting that there is a novel virus close to but different from known toroviruses. The genome sequence of this novel virus was obtained by assembling the torovirus-related reads from two rounds of metagenomic sequencing of *H. rufipes* ticks from Bangali, and gaps were filled by gap-filling PCR. The virus was named BanToV according to the location from where the ticks were collected. The complete genome of BanToV was 28,847 nt in length and shared 83% nt identity with the Brene virus strain P138/72, which was identified from *Equus caballus* in Switzerland (Table 1). The results of phylogenetic analysis based on the full genome sequence and the aa sequences of ORFs (ORF1a, S1, S2, M, HE, and N) of BanToV further suggest that BanToV is a novel member of the genus *Torovirus* in the family *Tobaniviridae* (Figure 3D and Supplementary figure S3).

### Prevalence and abundance of viruses in individual ticks

The substantial infection rates of the identified IFTV/MATV, BLTV4, LMTV, and BanToV were investigated among the tick individuals using the bead-based assays (Table S5). Among the remaining 336 ticks, one *H. truncatum* tick (0.29%) from Mbalambala was positive for IFTV or MATV and was further confirmed to be MATV by Sanger sequencing (data not shown). LMTV was detected in seven ticks (2.08%) from the four locations, with the highest infection rate (8.33%) among *H. rufipes* ticks from Balguda. BanToV was detected in 37 (11.01%) ticks; the positive rate was higher than that of the other tested viruses. The infection rates of BanToV in *H. truncatum* (32.58%) and *H. rufipes* (46.15%) ticks from Iftin (33.33%) were higher than those in *H. truncatum* (2.38%) and *H. rufipes* (4.16%) ticks from Bangali (2.89%). However, BLTV4 was not detected in any tick.

**Figure 4.**
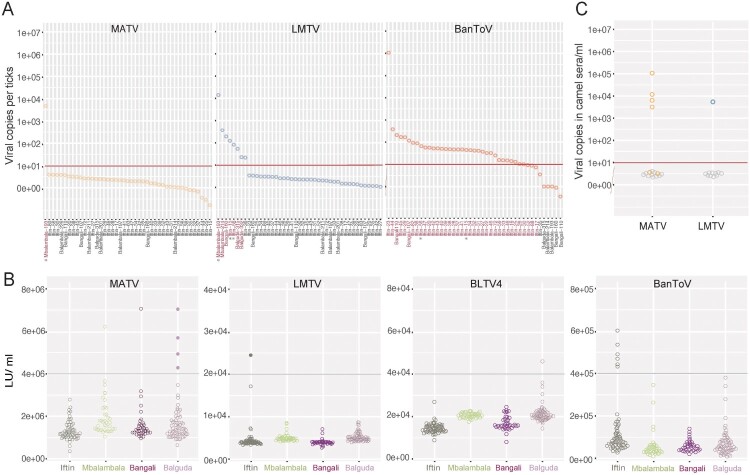
Prevalence of MATV, LMTV, BLTV4, and BanToV in each tick and camel serum sample. (A) The viral RNA copies in tick individuals are detected by qRT-PCR. Quantification result of 43 ticks, including all viral RNA-positive ticks (red) and several randomly selected viral RNA-negative ticks (black) detected on bead-based assays, are shown. The co-infection events are indicated by asterisks. (B) Seroprevalence of MATV, LMTV, BLTV4, and BanToV in camels. The luciferase activity (LU/mL) of each type of virus found in each camel serum sample examined by LIPS assay are shown as circles and distinguished by different colours according to the sampling locations. The cut-off values for each virus are indicated by black lines. The camel serum samples positive for both viral RNA and antibodies are shown in solid dots. (C) qRT-PCR detection of MATV and LMTV RNA copies in the seropositive samples. Ten virus copies are set as the threshold value and are indicated by red line as previously described [46]. Quantification of selected viruses in each individual tick and camel serum sample is performed using the ggplot2 package in R studio [35].

The viral positive rates shown on bead-based assays were further supported by the results of qRT-PCR, conducted for all viral RNA-positive ticks and randomly selected viral RNA-negative ticks (Figure 4A). The presence of MATV in one tick shown on bead-based assay was also confirmed by qRT-PCR. Eight and 34 ticks were positive for LMTV and BanToV, respectively. In addition, BLTV4 was not detected in the 43 ticks by qRT-PCR. Viral loads in tick individuals were also determined by qRT-PCR. The viral load of MATV detected in one tick was 6.2 × 10^3^ copies, while that of LMTV varied from 1.72 × 10^1^–2.69 × 10^4^ copies per tick. The load of BanToV in one tick was measured as 1.02 × 10^6^ copies; in other ticks, the loads were less than 5.12 × 10^2^ copies.

“Co-infection” events, i.e. detection of RNA of more than one virus in one tick, were noted in three *H. truncatum* ticks. Two ticks from Iftin (tick 15 and 56) exhibited low viral loads of LMTV and BanToV, while one tick from Mbalambala (tick 193) showed high viral copies of MATV and LMTV (Figure 4A). These results improved our understanding of the abundance and complexity of viruses vectored by a single tick.

Serological evidence and detection of viremia suggesting virus transmission between ticks and camels

Since ticks were collected from camels, they were suspected to have been exposed to or were infected with these viruses identified from ticks. The antibodies against NP of the four different viruses were detected in serum samples of camels using LIPS assay (Figure 4B and Table S6), which could detect both IgM and IgG responses [38]. Serum samples from six camels (6/200, 3%) were positive for antibodies against MATV, including one from Mbalambala (1/38, 2.63%), one from Bangali (1/39, 5.12%), and four from Balguda (4/63, 6.25%). Serum samples from only one camel from Iftin (1/59, 1.69%) were positive for antibodies against MATV, while those from one camel from Balguda (1/64, 1.56%) was positive for antibodies against BLTV4. A high serological rate specific to BanToV was observed in six of 59 (10.16%) camels from Iftin, thereby indicating BanToV infection. The results provided serological evidence that camels were exposed to TBVs in Kenya.

TBV infection might also have caused viremia in addition to the antibody response, which could be related to detectable viral RNA in the blood sample. Subsequently, antibody-positive serum samples were used for viral RNA detection by qRT-PCR, and 10 negative samples were randomly selected as controls. As expected, viral RNA was not detected in any of the 10 antibody-negative serum samples (data not shown). Four of the six anti-MATV-positive serum samples from the camels in Balguda were positive for MATV RNA with a viral load of 9.42 × 10^2–^4.43 × 10^4^ copies/mL (Figure 4B and C). The only LMTV-seropositive camel also had viremia with viral loads of 2.12 × 10^3^/mL was shown (Figure 4B and C). BLTV4 and BanToV RNA were not detected in the respective antibody-positive samples. These results further provided evidence of TBV infection in camels and indicated the transmission of viruses between ticks and camels.

## Discussion

This study reported the metagenomic profiles of *H. truncatum, H. rufipes,* and *H. dromedarii* ticks collected from camels, showing a diverse viral population related to at least 25 different viruses belonging to 11 viral families in Kenya. Viruses of the families *Flaviviridae*, *Phenuiviridae*, and *Chuviridae* were abundant in tick samples, consistent with the results of previous studies that viruses of these families were identified in tick species from countries in Europe, Asia, and Latin America [13,14,39]. This finding suggests that the families *Flaviviridae*, *Phenuiviridae*, and *Chuviridae* have spread in other countries worldwide and can be found in ticks. Five viruses, including three novel viruses (IFTV, MATV, and BanToV) and new strains of two known viruses (BLTV4 and LMTV), were detected in high abundance in tick pools and were obtained to determine their genome sequences. IFTV and MATV are unassigned viruses belonging to the family *Phenuiviridae* of the order Bunyavirales. Unlike the typical virus belonging to the family *Phenuiviridae*, such as severe fever with thrombocytopenia syndrome virus, which contains three RNA segments (L, M, and S), the M segments of IFTV and MATV were not identified from the sequencing data. The lack of the M segment was also observed among novel viruses such as BLTV1, Changping tick virus 1, Tacheng tick virus 2, and Lihan tick virus, probably due to the high divergence of M segment sequences from other known viruses [10]. BLTV4 was previously discovered in *H. asiaticum* ticks in China and *R. sanguineus* ticks in Trinidad and Tobago [40,41]. LMTV was identified in *H. anatolicum* ticks from Russia in 2018 according to the viral sequence information deposited in GenBank (MN542376). The new strains of BLTV4 and LMTV identified in this study provided evidence of the presence of unclassified flavi-like viruses and Chuviruses in Africa, suggesting a wide distribution of these TBVs worldwide. Toroviruses are important causative pathogens of diarrhea, which were mostly discovered in faecal samples of humans and domestic animals [42,43]. Identification of BanToV in camel-derived ticks suggest the presence of a novel pathogen that causes zoonotic diseases in Kenya. The viromes in this study led to the discovery of novel viruses and new viral strains, enhancing the understanding of the novel TBVs and their phylogenetic diversities in Kenya and expanding the knowledge on the global distribution of TBVs.

Subsequently, this study investigated the infection rates of MATV, BLTV4, LMTV, and BanToV in tick individuals rather than in tick groups. BanToV had the highest infection rate among ticks, followed by LMTV and MATV. Based on the sampling locations, LMTV was more widely distributed than the other tested viruses as it was detected in ticks from four sites. Generally, the infection rates of BanToV, LMTV, and MATV were higher in *H. truncatum* ticks than in *H. rufipes* ticks. Viruses were not detected in any *H. dromedarii* ticks, probably because of the limited sample size. The substantial infection rates in tick individuals were also supported by the results of qRT-PCR, which measured the viral RNA copies and promoted the understanding of viral loads in each viral RNA-positive tick.

**Figure 5.**
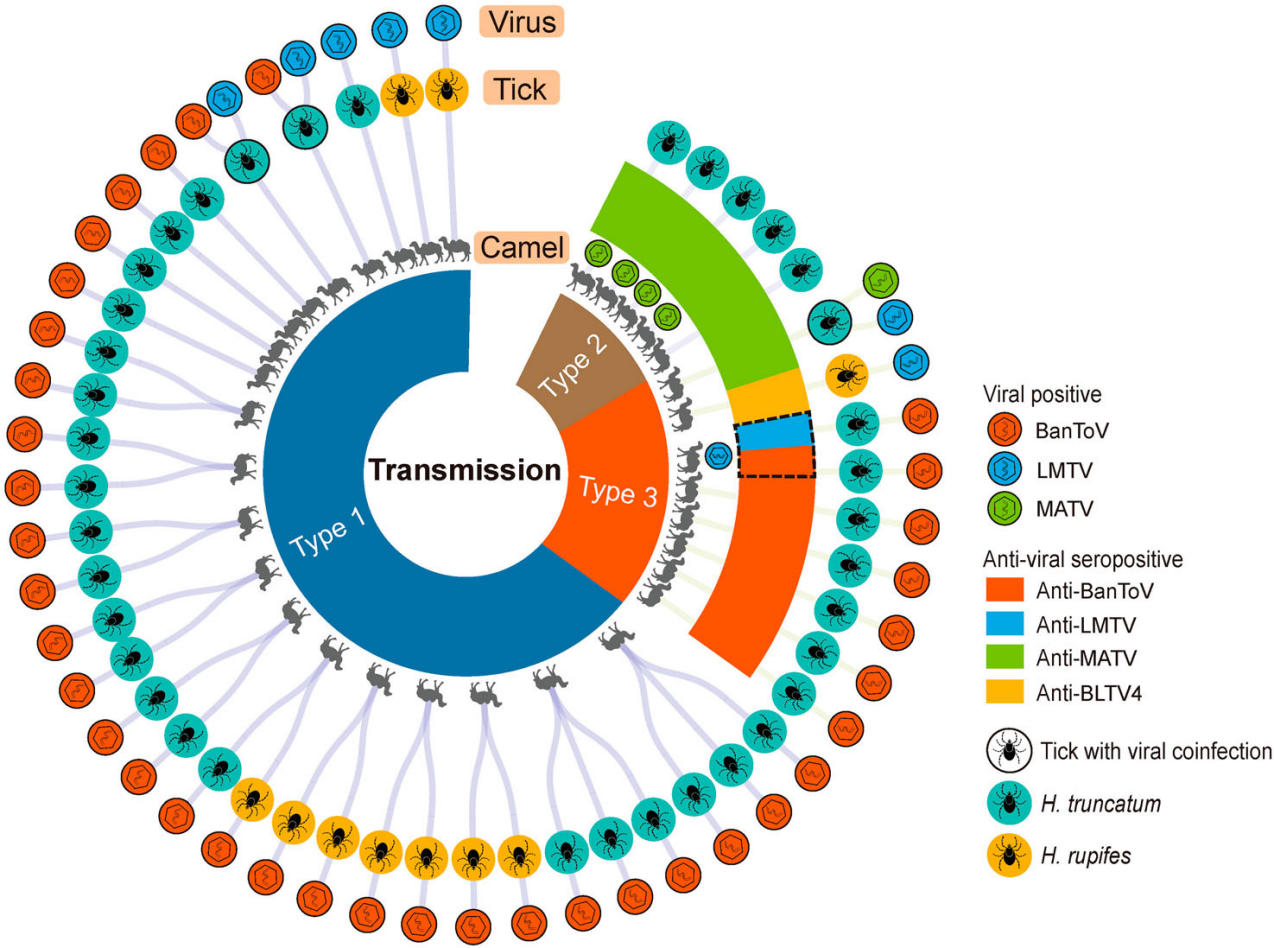
Patterns of viral transmission between ticks and camels in Kenya. Three transmission patterns (types 1, 2, and 3) are characterized based on the results of viral RNA detection in ticks and camel serum samples and anti-viral antibody detection in camels. The transmission correlations of the camels, ticks, and identified viruses are shown as shaded lines. Each camel and tick that positive for viral RNA is labelled with the diagram of viral particles. Camels positive for the antibody against viruses are indicated by coloured boxes. Tick species can be distinguished by their different colours. Virus species and specific antibodies are indicated by different colours. Red, BanToV; blue, LMTV; green, MATV. A single tick co-infected with more than one virus is indicated by a circular outline. The camel whose antibody responds to two different viruses is labelled with a dashed outline. The landscape of the viral transmission correlations is plotted and adjusted using d3.js (https://d3js.org).

Ticks can transmit viruses to animal hosts through blood feeding. Therefore, the infection rates and serological responses against MATV, BLTV4, LMTV, and BanToV were investigated in camels with direct sampling correlations with the tick samples. Viral RNA was detected in serum samples from five camels (four for IFTV/MATV and one for LMTV), and antibodies against MATV, LMTV, BLTV4, and BanToV were also found despite the small number of camels. Among them, five camels (four for MATV and one for LMTV) had both viral RNA and antibodies against each of the abovementioned viruses. In addition, the co-infection observed in three *H. truncatum* ticks suggested the complexity of viruses vectored by a single tick. Although co-infection was not observed in camels by viral RNA detection, one camel from Iftin had antibodies against both MATV and BanToV, indicating that co-infection with the two viruses might have occurred in this camel (Figure 5). These results demonstrated that MATV, BLTV4, LMTV, and BanToV were possibly acquired by exposure to the blood of infected animals.

As the ticks and serum samples from camels were collected with clearly recorded tick–camel correlations, the patterns of transmission of viruses between ticks and camels, including BanToV, LMTV, MATV, and BLTV4, were characterized based on the results of RNA-seq and antibody detection (Figure 5). By matching the tick indivaduals with the correlated camels, we found that there could be three types of correlations between ticks with virus infection and camels with response to viruses. First, serum samples from camels were negative for both viral RNAs and antibodies, whereas viral RNA was detected in the ticks collected from correlated camels (Figure 5, type 1). This was observed in 33 ticks collected from 20 camels, among which 28 ticks (90.9%) were positive for BanToV, three ticks (9.1%) were positive for LMTV, and two ticks (6.1%) exhibited BanToV and LMTV co-infection. This finding suggested that the virus vectored by ticks did not infect these camels or that virus replication did not possibly occur in camels. Second, viral RNA was not detected in ticks, whereas viral RNA and/or antibodies were detected in camels (Figure 5, type 2). Five camels had an antibody response to MATV, and four of them were positive for MATV RNA. This result suggested that these camels were infected with MATV and generated antibodies against this virus. However, the five ticks collected from these camels were not infected with MATV, probably because MATV was not spread from camel to tick or virus replication was not initiated in these ticks at the time of sampling. Third, the virus was detected in tick individuals, and viral RNA and/or antibodies were detected in correlated camels (Figure 5, type 3). Five camels had antibodies against BanToV, whereas the five ticks collected from these camels were infected with BanToV. One camel was positive for LMTV RNA and had antibodies against both LMTV and BanToV, while the two ticks collected from this camel were positive for BanToV RNA. This finding suggests that substantial BanToV transmission occurred between the ticks and camels. One camel had an antibody response to MATV, while the tick from this camel was positive for MATV and was co-infected with LMTV, suggesting the occurrence of MATV transmission between ticks and camels. Moreover, one camel had antibodies against BLTV4, but the tick from this camel was infected with LMTV instead of BLTV4. The different transmission patterns could be related to the differential competence of tick vectors to transmit viruses. Based on the above patterns, we found that BanToV had a high transmission possibility. In total, 37 ticks from 25 camels were positive for BanToV RNA, among which six camels (24.0%) had antibody exposure to the virus, suggesting the frequent transmission of BanToV. BanToV is likely derived from camels, as other toroviruses are related to the development of zoonotic diseases. The high prevalence among ticks also suggest the ability of ticks to spread BanToV. Despite the first identification of chuvirus, flavi-like virus, and novel phenuivirus vectored by ticks a few years ago, the infectivity rate of these viruses to animals or humans is poorly understood. Seven ticks were positive for LMTV, but they were not derived from camels with LMTV infection and antibody response. Six camels had antibodies against MATV, four of which were positive for MATV RNA; however, only one tick from one of these camels had MATV infection. One camel had an antibody against BLTV4, but BLTV4 RNA was not detected in any of the ticks. These results suggest the presence of competent viral vectors for LMTV, MATV, and BLTV4 and the viral transmission between ticks and camels in Kenya. We speculate that the high viral loads vectored by ticks promote virus transmission to hosts. However, the virus copies in each tick were not significantly correlated with the viral loads and antibody responses in the tick-infected camels, which might be affected by the sampling time and sample size. Therefore, continuous investigations on an increasing size of samples from ticks and tick-derived hosts would further clarify the substantial transmission and reveal potential risks from the infection of TBVs.

Camels are one of the major livestock animals important for agriculture and tourism in Kenya and could be a natural reservoir of viral pathogens [44,45]. Camel handlers, such as livestock farmers, traders, and butchers, may be at high risk of infectious diseases caused by camel-derived viruses. Therefore, camels infected with LMTV, MATV, BLTV4, and BanToV may also pose threats by spreading the virus to humans. We believe that the current study initiated an elaborate investigation of the viral transmission correlations between tick vectors and animal hosts and their impact in human living activities. One limitation of this study is that the investigation of TBV transmission was performed based on the results of a single survey in Kenya using a small number of samples. There is still an urgent need to perform follow-up investigations in the hotspot area of TBVDs, such as Kenya, on a large number of samples including humans, animal hosts, and ticks and the definite records of the sampling relationships between the individuals, which will enhance the understanding of the transmission origin between ticks and hosts and possible emergence of new virus strains.

In summary, this study revealed the viromes of three *Hyalomma* tick species in Kenya; identified LMTV, MATV, BLTV4, and BanToV as potential viral pathogens; characterized their transmission between ticks and camels; and consequently suggested substantial threats from infection. These findings may guide the prevention and control of TBV-related infectious diseases in Kenya and other African countries.

## Supplementary Material

Supplementary_figure_3.jpg

Supplementary_figure_2.jpg

Supplementary_figure_1.jpg

Clean_supplementary_tables.docx

Supplemantary_data_kenya_camel_tick_viruses_20210715_for_EMI_1_.docx
