# Supplementary figures and images for: Viromes and surveys of RNA viruses in camel-derived ticks revealing transmission patterns of novel tick-borne viral pathogens in Kenya
Source: Emerg Microbes Infect. 2021 Oct 17;10(1):1975–87. doi: 10.1080/22221751.2021.1986428 (PMC8525980; doi:10.1080/22221751.2021.1986428)

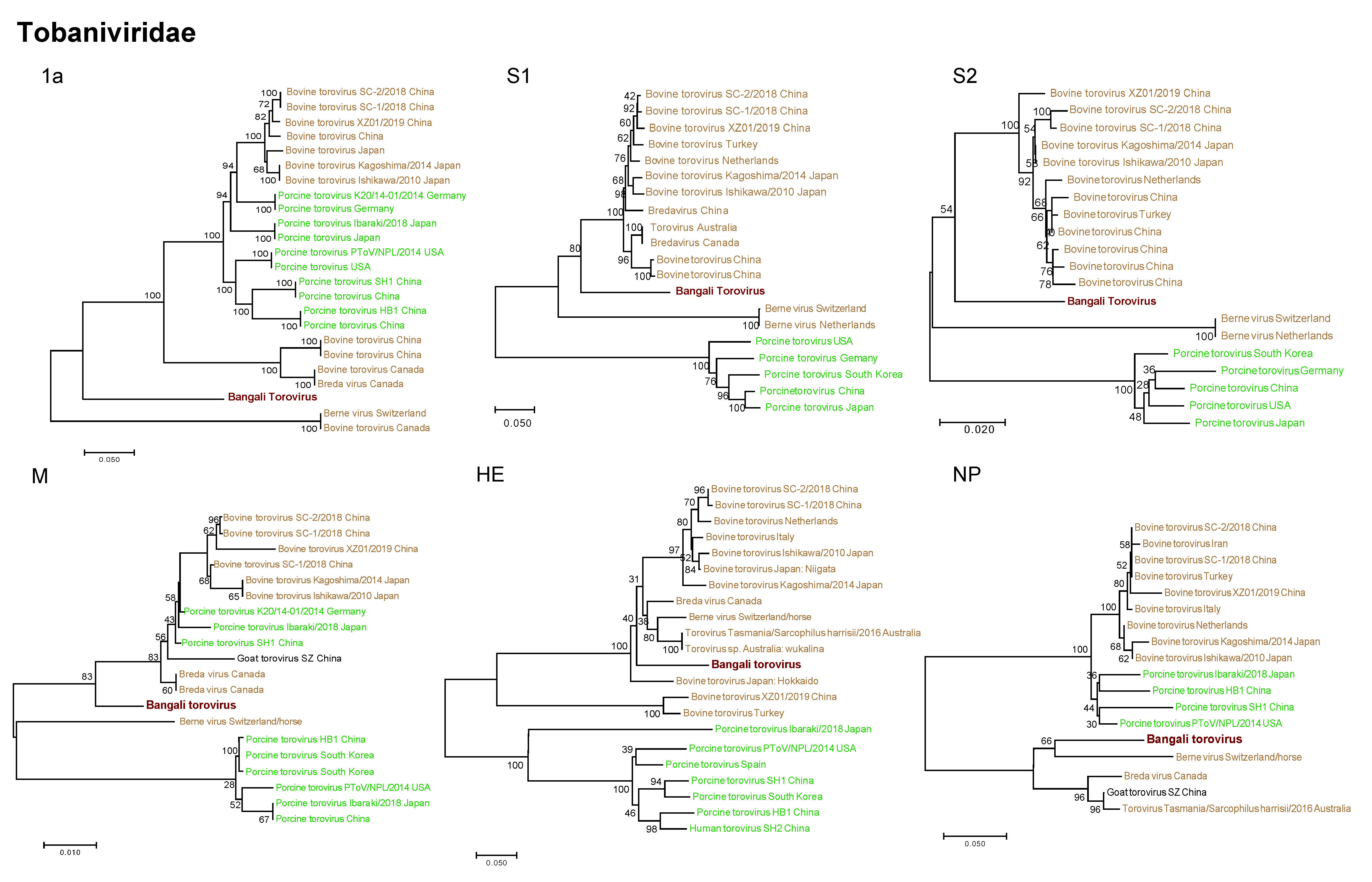

Supplement: Supplementary_figure_3.jpg [file TEMI_A_1986428_SM4979.jpg]

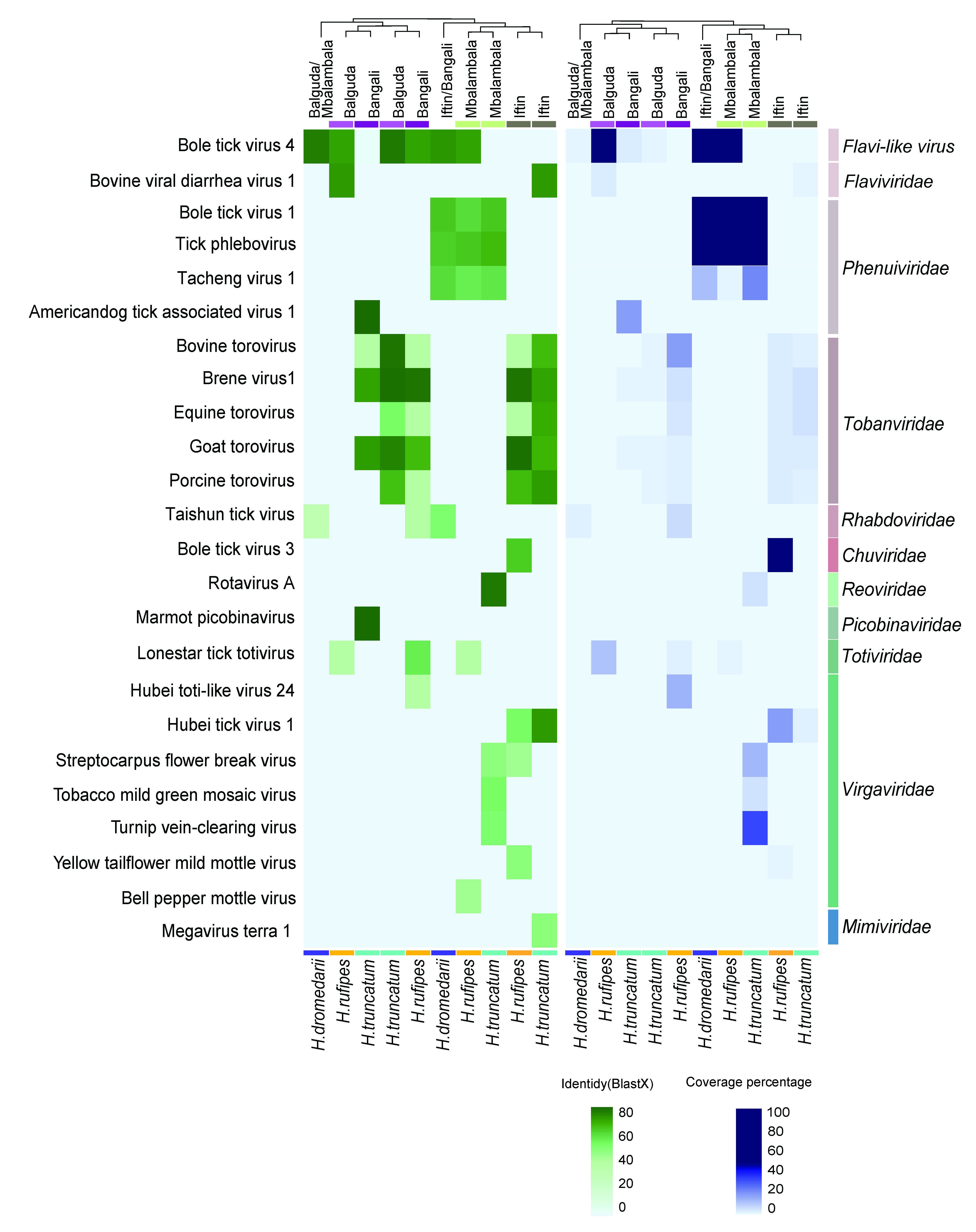

Supplement: Supplementary_figure_2.jpg [file TEMI_A_1986428_SM4978.jpg]

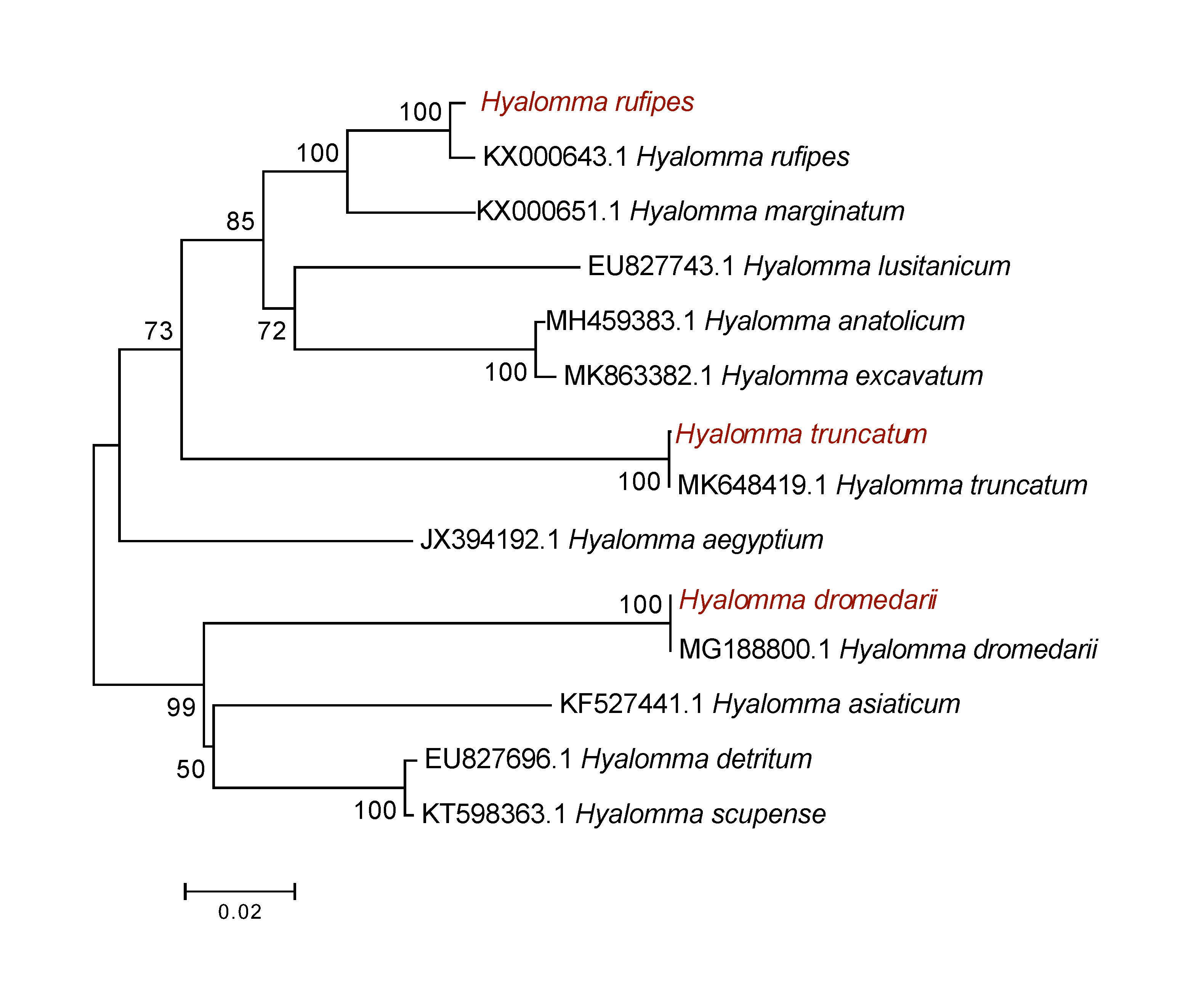

Supplement: Supplementary_figure_1.jpg [file TEMI_A_1986428_SM4977.jpg]
