# Supplementary material for: Viromes and surveys of RNA viruses in camel-derived ticks revealing transmission patterns of novel tick-borne viral pathogens in Kenya
Source: Emerg Microbes Infect. 2021 Oct 17;10(1):1975–87. doi: 10.1080/22221751.2021.1986428 (PMC8525980; doi:10.1080/22221751.2021.1986428)
Supplement: Clean_supplementary_tables.docx [file TEMI_A_1986428_SM4976.docx]

**Supplementary Tables**

**Table S1.** Sampling information of ticks and camels in Kenya

| Location county | Locations | Locality GPS | No. camels | Tick species | No. ticks |
| --- | --- | --- | --- | --- | --- |
| Garissa | Iftin | 0.553971°S, 39.951464°E | 60 | *H. truncatum* | 94 |
|  |  |  |  | *H. rufipes* | 18 |
|  |  |  |  | *H. dromedarii* | 8 |
|  | Balambala | 0.029668°S, 39.387576°E | 39 | *H. truncatum* | 51 |
|  |  |  |  | *H. rufipes* | 22 |
|  |  |  |  | *H. dromedarii* | 5 |
| Tana River | Bangali | 0.824663°S, 39.006526°E | 41 | *H. truncatum* | 47 |
|  |  |  |  | *H. rufipes* | 29 |
|  |  |  |  | *H. dromedarii* | 8 |
|  | Balguda | 1.431621°S, 39.834425°E | 60 | *H. truncatum* | 69 |
|  |  |  |  | *H. rufipes* | 29 |
|  |  |  |  | *H. dromedarii* | 16 |
| **Total** |  |  |  |  | **396** |

**Table S2.** Summary details of each RNA sequencing pools

| Pools | Tick species | Tick host | Collection location | No. individuals | No.Raw Reads |
| --- | --- | --- | --- | --- | --- |
| 1 | *H. truncatum* | Camel | Iftin | 5 | 35,504,026 |
| 2 | *H. rufipes* | Camel | Iftin | 5 | 36,663,058 |
| 3 | *H. truncatum* | Camel | Balambala | 5 | 32.550,160 |
| 4 | *H. rufipes* | Camel | Balambala | 5 | 34,666,484 |
| 5 | *H. truncatum* | Camel | Bangali | 5 | 38.657,797 |
| 6 | *H. rufipes* | Camel | Bangali | 5 | 33,808,271 |
| 7 | *H. truncatum* | Camel | Balguda | 5 | 34,127,437 |
| 8 | *H. rufipes* | Camel | Balguda | 5 | 43,927,631 |
| 9 | *H. dromedarii* | Camel | Iftin/Bangali | 10 | 37,920,458 |
| 10 | *H. dromedarii* | Camel | Balambala/Balguda | 10 | 41,209,419 |

**Table.S3** Nested-PCR and Beads-based detection primer and probe

| Viruses |  | Seq |
| --- | --- | --- |
| IFTV and BATV | F-primer | ATCAGGAAATGCGACGAGC |
|  | R-primer | GGGTGCCTAAACTTGGTGC |
|  | R'-primer | GTCGGAGATATGTCATGGAGTGT |
|  | F-primer* | GTTACAGGGTTAGAAACTTAGATGC |
|  | R-primer* | TCGTCTGCGGTGGCTACTCTG ( 5`Cy3) |
|  | Probe* | ACGTATGGTGATGGGTGCCCGGTGTTCACAACACTC (5`NH2 C12) |
| BLTV4 | F-primer | CACTCTGTATGCGACTGTTTGA |
|  | R-primer | GCAGGTCCTCACGAAAGCAG |
|  | R'-primer | ATTCAGGAGCAGGTCAGAAAC |
|  | F-primer* | AGCTTCTATGACAAAGAGCAC |
|  | R-primer* | CTACCTCAAAGGTGAGGTAGTC ( 5`Cy3) |
|  | Probe* | TGTGTTTCACAAGGCTTGGCTTCGTTTTCACCAATC (5`NH2 C12) |
| LMTV | F-primer | TACATACATCAGATCAAAGTTTGCA |
|  | R-primer | ATGAGTATGGATCTACCATCAG |
|  | R'-primer | TTGCAGAACGTGGACAAGCAT |
|  | F-primer* | GATCAATGAAGAGTCCCACCTG |
|  | R-primer* | ACTCTGTTGGCACATGCTAC ( 5`Cy3) |
|  | Probe* | ATGGCTTATCAAACACTGATGTGTGTGCTGTTATAC (5`NH2 C12) |
| BanToV | F-primer | GTCAAGCATGTGGCAATCTC |
|  | R-primer | ACCGCATTAATGCAGGAC |
|  | R'-primer | GGTCTACTCATATACCTAAC |
|  | F-primer* | GTCAAGCATGTGGCAATCTC |
|  | R-primer* | ACCGCATTAATGCAGGAC ( 5`Cy3) |
|  | Probe* | GGTCTACTCATATACCTAACTATAAACAAATTGGTC (5`NH2 C12) |

*primer and probe designed for Beads-based assay

**Table S4.** Viral sequences identified in the tick pools by RNAseq

| **Family** | **Genus** | **Reference virus** | **The number of virus-related read counts** | | | | | | | | | | **AA**  **Identity**  **(%)** |
| --- | --- | --- | --- | --- | --- | --- | --- | --- | --- | --- | --- | --- | --- |
|  |  |  | **Iftin** | | **Mbalambala** | | **Bangali** | | **Balguda** | | **Iftin/**  **Bangali** | **Mbalambala**  **/Balguda-** |  |
|  |  |  | *H. trun* | *H. rufi* | *H. trun* | *H. rufi* | *H. trun* | *H. rufi* | *H. trun* | *H. rufi* | *H. drom* | *H. drom* |  |
| *Flaviviridae* | Pestivirus | Bole tick virus 4 |  |  |  | 2888 | 2463 |  | 14 | 17155 | 19992 | 20 | 92-94 |
|  |  | Bovine viral diarrhea virus 1 | 3 |  |  |  |  |  |  | 10 |  |  | 72 |
| *Tobaniviridae* | Torovirus | Bovine torovirus, BToV | 900 |  |  |  | 2 | 200 | 11 |  |  |  | 57-84 |
|  |  | Brene virus 1 | 12 | 8 |  |  | 34 | 625 | 8 |  | 6 |  | 60-80 |
|  |  | Goat torovirus | 324 | 9 |  |  | 23 | 180 | 28 |  |  |  | 95-96 |
|  |  | Equine toroviru | 324 | 9 |  |  |  | 170 | 14 |  |  |  | 84-88 |
|  |  | Porcine torovirus | 21 | 56 |  |  |  | 88 | 7 |  |  |  | 82-90 |
| *Virgaviridae* | Hubei tick virus 1 | Hubei tick virus 1 | 12 | 305 |  |  |  |  |  |  |  |  | 38-86 |
|  | Tobamovirus | Turnip vein-clearing virus |  |  | 80 |  |  |  |  |  |  |  | 65 |
|  |  | Streptocarpus flower break virus |  | 6 | 26 |  |  |  |  |  |  |  | 54 |
|  |  | Hubei toti-like virus 24 |  |  |  |  |  | 16 |  |  |  |  | 55-61 |
|  |  | Yellow tailflower mild mottle virus |  | 12 |  |  |  |  |  |  |  |  | 60 |
|  |  | Tobacco mild green mosaic virus |  |  | 7 |  |  |  |  |  |  |  | 65 |
|  |  | Bell pepper mottle virus |  | 5 |  |  |  |  |  |  |  |  | 80 |
| *Phenuiviridae* | Uukuvirus | American dog tick associated virus 1 |  |  | 14604 | 3421 |  |  |  |  | 145 |  | 72 |
|  | unclassified | Tick phlebovirus |  |  | 1394 | 2453 |  |  |  |  | 421 |  | 78-80 |
|  |  | Bole tick virus 1 |  |  | 15475 | 28838 |  |  |  |  | 900 |  | 73-82 |
| *Nairoviridae* | Orthonairovirus | Tacheng virus 1 |  |  |  |  | 12 |  |  |  |  |  | 92-98 |
| *Rhabdoviridae* | unclassified | Taishun tick virus |  |  |  |  |  | 130 |  |  | 4 | 7 | 59-79 |
| *Chuviridae* | Mivirus | Liman tick virus |  | 6075 |  |  |  |  |  |  |  |  | 90-92 |
| *Reoviridae* | Rotavirus | Rotavirus A |  |  | 186 |  |  |  |  |  |  |  | 89-100 |
| *Totiviridae* | unclassfied | Lonestar tick totivirus |  |  |  | 22 |  | 8 |  | 123 |  |  | 38-57 |
| *Picobirnaviridae* | Picobirnavirus | Marmot picobinavirus |  |  |  |  | 1 |  |  |  |  |  | 98-100 |
| *Mimiviridae* | unclassified | Megavirus terra1 | 3 |  |  |  |  |  |  |  |  |  | 95-100 |
| **Total** |  |  | **1599** | **6485** | **31772** | **37622** | **2535** | **1417** | **82** | **1737** | **21468** | **27** |  |

*H. trun*, *H. truncatum; H. rufi*, *H. rufipes; H. drom*, *H. dromedarii.*

**Table S5.** Substantial infection rates of IFTV/MATV, BLTV4, LMTV and BanToV in tick individuals

| Collection regions | Tick species | No. of ticks | IFTV/ MATV | BLTV4 | LMTV | BanToV |
| --- | --- | --- | --- | --- | --- | --- |
| Iftin | Subtotal | 105 | 0 | 0 | 2 (1.90%) | 35 (33.33%) |
|  | *H. truncatum* | 89 | 0 | 0 | 2 (2.24%) | 29 (32.58%) |
|  | *H. rufipes* | 13 | 0 | 0 | 0 | 6 (46.15%) |
|  | *H. dromedarii* | 3 | 0 | 0 | 0 | 0 |
| Mbalambala | Subtotal | 63 | 1 (1.58%) | 0 | 2 (3.17%) | 0 |
|  | *H. truncatum* | 46 | 1 (2.17%) | 0 | 1 (2.17%) | 0 |
|  | *H. rufipes* | 17 | 0 | 0 | 1 (5.8%) | 0 |
|  | *H. dromedarii* | 0 | N/A | N/A | N/A | N/A |
| Bangali | Subtotal | 69 | 0 | 0 | 1 (1.44%) | 2 (2.89%) |
|  | *H. truncatum* | 42 | 0 | 0 | 1 (2.38%) | 1 (2.38%) |
|  | *H. rufipes* | 24 | 0 | 0 | 0 | 1 (4.16%) |
|  | *H. dromedarii* | 3 | 0 | 0 | 0 | 0 |
| Balguda | Subtotal | 99 | 0 | 0 | 2(2.02%) | 0 |
|  | *H. truncatum* | 64 | 0 | 0 | 0 | 0 |
|  | *H. rufipes* | 24 | 0 | 0 | 2 (8.33%) | 0 |
|  | *H. dromedarii* | 11 | 0 | 0 | 0 | 0 |
| **Total** |  | **336** | **1 (0.29%)** | **0** | **7 (2.08%)** | **37 (11.01%)** |

**Table S6.** Seropositivity rate of MATV, BLTV4, LMTV and BanToV among camel populations

| Collections | No. of test | MATV | BLTV4 | LMTV | BanToV |
| --- | --- | --- | --- | --- | --- |
| Iftin | 59 | 0 | 0 | 1 (1.69%) | 6 (10.16%) |
| Mbalambala | 38 | 1 (2.63%) | 0 | 0 | 0 |
| Bangali | 39 | 1 (2.56%) | 0 | 0 | 0 |
| Balguda | 64 | 4 (6.25%) | 1 (1.56%) | 0 | 0 |
| **Total** | **200** | **6 (3.00%)** | **1 (0.05%)** | **1 (0.05%)** | **6 (3.00%)** |
