# Supplementary material for: Viromes and surveys of RNA viruses in camel-derived ticks revealing transmission patterns of novel tick-borne viral pathogens in Kenya
Source: Emerg Microbes Infect. 2021 Oct 17;10(1):1975–87. doi: 10.1080/22221751.2021.1986428 (PMC8525980; doi:10.1080/22221751.2021.1986428)
Supplement: Supplemantary_data_kenya_camel_tick_viruses_20210715_for_EMI_1_.docx [file TEMI_A_1986428_SM4975.docx]

**Supplementary Methods**

***Identification of tick species by PCR***

DNA was extracted from a single tick using the AxyPrep Multisource Genomic Miniprep DNAkit (Axygen, USA) according to the manufacture’s instruction and was used to amplify the COX1 gene with specific primers (F: 5’-GGAGGATTTGGAAATTGATTAGTTCC-3’ and R: 5’- ACTGTAAATATATGATGAGCTCA-3’) (Barcode of Life Data System [http://www.barcodinglife.org]) using the KOD -Plus- NeoHigh Fidelity & efficient & fast DNA polymerase (TOYOBO, Japan). PCR products were sequenced by Sanger sequencing and compared with ixodid tick genome database in NCBI by BLASTn.

***Analyses of viral-related sequences from data of metagenomic sequencing***

The quality of the sequencing results was evaluated using the FastQC (version 0.11.9). All sequencing reads were first aligned with the ixodid tick genome database to filter out the tick-related reads. Subsequently, host-free reads were used for *de novo* assembly by Trinity (version 2.4.0) and subjected to alignment with non-redundant virus nucleotide and protein databases from NCBI using BLASTn, BLASTx, and DIAMOND (<http://ab.inf.uni-tuebingen.de/software/diamond>) [1]. Viral-related significant contigs with e-values lower than 1e-5 were retained, and the virome component composition was analyzed using MEGAN (version 5.11.3). The number of sequencing reads assembled into each contigs were calculated using Bowtie 2 (version 2.1.0) [2]. Statistical difference analysis of the total and proportions of eukaryotic, bacterial, and viral reads from the four locations and three tick species was performed by GraphPad Prism (version 8.0) with Wilcoxon test.

***Bead-based multiplex assay***

The Bead-based multiplex assay was established to detect multiple viral RNA vectored by a single tick in one reaction. First, clarified homogenates of a single tick were prepared as previously described [3]. Total RNA was extracted from the homogenates of a single tick using the automatic nucleic acid purification instrument (Novastar, Wuhan, China) according to the manufacturer’s instruction. Subsequently, RT-PCR was performed using the One Step PrimeScript RT-PCR Kit (TAKARA, Japan) according to the manufacturer’s instructions. Briefly, the reaction was carried out for 40 cycles in a 40µL volume of mixture containing 5 µL RNA extracted from a single tick, 10 µL 2 × One step RT-PCR buffer, 0.4 µL Ex Taq HS, 0.4 µL PrimeScript RT Enzyme Mix, 0.4 µL forward primer and 0.4 µL reverse primer specific to selected viruses (Table S3). Then, the RT-PCR products (5 µL) from each tick sample was hybridized with 50 µL mixed beads (NZK-LB-M1001-1005-XX, Novastar, Wuhan, China) coupled with oligonucleotide probes labeled by Cy3 (5000 beads of each type) at 42 °C for 90 min to achieve the specific binding of probe and amplicons. Finally, the hybridized products were used to detect the fluorescence of Cy3 from the probe using a biochemical luminescence detector NovaHT (Novastar, Wuhan, China), and different viral amplicons were distinguishable from each other in terms of the distinct biomarkers in the beads. The fluorescence value of negative control (RNase-free water) was set as threshold.

***qRT-PCR to detect viral loads***

To generate the RNA standards of each tested viruses, PCR was performed to amplify partial sequences of the target gene using the primer T7-F that the T7 promoter sequence (TAATACGACTCACTATAGGGAGA) fused with primer F at its 5’end and the primer R for respective virus (Table S3) using the cDNA from the virus-positive tick pool. PCR products were purified using Gel extraction kit (Omega, USA) and were then used as the template for RNA synthesis in vitro using T7 RNA polymerase (Beyotime, China) according to the manufacture’s instruction. RNA was purified and quantified using NanoDrop 1000 (Thermo Fisher Scientific, USA). To prepare the standard curve for qRT-PCR detection of each virus, RNA was ten-fold serially diluted using RNase-free ddH_2_O (TAKARA, Japan) to generate the RNA standards containing copies from 10^8^ to 10^1^. The qRT-PCR was performed with the RNA from tick individuals and the standards by using the One Step PrimeScript™ RT-PCR Kit (TAKARA, Japan) as described above.

***LIPS assays***

Different viral antigens fused with Ruc were harvested from HEK293T cells transfected with related recombinant plasmid, and then the light unit (LU) of each Ruc-viral antigen extraction was measured using Renilla-LumiTM Luciferase Assay Kit (Beyotine, China) on a GloMax 20/20 (Promega, USA). The LIPS assay for the antibody detection in camel serum samples was performed as previously described [4]. Briefly, 1 × 10^7^ LU of different Ruc-antigen extraction, 6 µL of 30% protein A/G beads (Thermo Fisher Scientific, USA) in PBS, and 100 µL 50-fold dilution of camel serum samples were added to each well of the 96-well plate and incubated at 4 °C for 4 h with gentle shaking. After incubation, the 96-well plate was subjected to shock centrifugation, and the supernatant was removed. The beads were washed with buffer A (50 mM Tris, pH 7.5, 100 mM NaCl, 5 mM MgCl_2_, 1% Triton X-100) for four times and with PBS twice before LU detection. The LU average of all the added 200 camel sera samples multiplied by three times the SD was set as the threshold as previously described [5].

**Reference**

1. Buchfink B, Xie C, Huson DH. Fast and sensitive protein alignment using DIAMOND. Nat Methods. 2015 Jan;12(1):59-60.

2. Langmead B, Salzberg SL. Fast gapped-read alignment with Bowtie 2. Nat Methods. 2012 Mar 4;9(4):357-9.

3. Shi CY, Beller L, Debouttel W, et al. Stable distinct core eukaryotic viromes in different mosquito species from Guadeloupe, using single mosquito viral metagenomics. Microbiome. 2019 Aug 28;7(1):121.

4. Zhang W, Zheng XS, Agwanda B, et al. Serological evidence of MERS-CoV and HKU8-related CoV co-infection in Kenyan camels. Emerg Microbes Infect. 2019;8(1):1528-1534.

5. Burbelo PD, Ching KH, Klimavicz CM, et al. Antibody profiling by Luciferase Immunoprecipitation Systems (LIPS). J Vis Exp. 2009 Oct 7(32):1549.
